# Effects of calcium channel blockers on glucose tolerance, inflammatory state, and circulating progenitor cells in non-diabetic patients with essential hypertension: a comparative study between Azelnidipine and amlodipine on glucose tolerance and endothelial function - a crossover trial (AGENT)

**DOI:** 10.1186/1475-2840-10-79

**Published:** 2011-09-10

**Authors:** Kosuke Fukao, Kazunori Shimada, Makoto Hiki, Takashi Kiyanagi, Kuniaki Hirose, Atsumi Kume, Hiromichi Ohsaka, Rie Matsumori, Takeshi Kurata, Tetsuro Miyazaki, Hiroyuki Daida

**Affiliations:** 1 Department of Cardiovascular Medicine, Juntendo University School of Medicine, 2-1-1, Hongo, Bunkyo-ku, Tokyo 113-8421, Japan

## Abstract

**Background:**

Hypertension is associated with impaired glucose tolerance and insulin resistance. Medical treatment that interferes with various steps in the renin-angiotensin system improves glucose tolerance and insulin resistance. However, it remains unclear if long-acting calcium channel blockers (CCBs) such as azelnidipine and amlodipine affect glucose tolerance and insulin resistance in clinical practice.

**Methods:**

Seventeen non-diabetic patients with essential hypertension who had controlled blood pressure levels using amlodipine (5 mg/day) were enrolled in this study. After randomization, either azelnidipine (16 mg/day) or amlodipine (5 mg/day) was administered in a crossover design for 12-weeks. At baseline and the end of each CCB therapy, samples of blood and urine were collected and 75 g oral glucose tolerance test (OGTT) was performed. In addition, hematopoietic progenitor cells (HPCs) were measured at each point by flow cytometry and endothelial functions were measured by fingertip pulse amplitude tonometry using EndoPAT.

**Results:**

Although blood pressure levels were identical after each CCB treatment, the heart rate significantly decreased after azelnidipine administration than that after amlodipine administration (*P *< 0.005). Compared with amlodipine administration, azelnidipine significantly decreased levels of glucose and insulin 120 min after the 75 g OGTT (both *P *< 0.05). Serum levels of high-sensitivity C-reactive protein (*P *= 0.067) and interleukin-6 (*P *= 0.035) were decreased. Although endothelial functions were not different between the two medication groups, the number of circulating HPCs was significantly increased after azelnidipine administration (*P *= 0.016).

**Conclusions:**

These results suggest that azelnidipine treatment may have beneficial effects on glucose tolerance, insulin sensitivity, the inflammatory state, and number of circulating progenitor cells in non-diabetic patients with essential hypertension.

## Background

Type 2 diabetes mellitus (DM) and hypertension are strong risk factors for coronary artery disease (CAD) [1]. In addition, postprandial hyperglycemia and hyperinsulinemia are considered to be risk factors for atherosclerotic disease [2,3]. Hypertension is associated with impaired glucose tolerance and insulin resistance, resulting in the development of DM in hypertensive patients [4,5]. If hypertension and DM coexist, the risk of cardiovascular disease increases by 2- to 3-fold [6]. Therefore, medications for preventing new-onset of DM as well as for treatment of hypertension are important in non-diabetic patients with hypertension. A recent meta-analysis demonstrated the association between types of antihypertensive agents and incidence of new-onset of DM [7]. The findings suggested that the association between anti-hypertensive agents and incident of DM was lowest for angiotensin-converting-enzyme (ACE) inhibitors and angiotensin-receptor blockers (ARBs) [7]. However, the anti-diabetic effect of calcium channel blockers (CCBs) is unclear.

Azelnidipine, a novel long-acting dihydropyridines-based CCB, reduces blood pressure without increasing the heart rate in patients with hypertension [8]. Azelnidipine has been reported to exhibit organ-protective effects, including anti-remodeling after myocardial infarction [9], renoprotection [10], and retarding atherosclerotic plaque progression [11]. In addition, azelnidipine has several unique basic and clinical effects, including inhibition of tumor necrosis factor (TNF)-α-induced interleukin (IL)-8 expression in human umbilical vein endothelial cells by blocking the generation of nicotinamide adenine dinucleotide phosphate oxidase-mediated reactive oxygen species [12]; reduction in urinary protein secretion and urinary 8-hydroxydeoxyguanosine and liver-type fatty acid binding protein (L-FABP) levels [10]; and reduction in circulating advanced glycation end-product (AGE) and soluble form of AGE [13]. Recent experimental studies demonstrated that azelnidipine improved glucose intolerance and lowered the risk of hyperglycemia-induced metabolic disorders in diabetic mice [14,15]. However, its effect on glucose tolerance and insulin sensitivity in the clinical practice has not been studied.

We hypothesized that azelnidipine administration could improve glucose tolerance and insulin levels in non-diabetic patients with essential hypertension. We examined the levels of blood glucose and insulin after the 75 g oral glucose tolerance (OGTT), lipids, inflammatory markers, circulating number of progenitor cells, and endothelial functions after administration of two CCBs, azelnidipine and amlodipine in a prospective randomized crossover study.

## Methods

### Subjects

Eighteen non-diabetic patients with essential hypertension were enrolled in this study. All subjects were administered amlodipine 5 mg once daily and had controlled blood pressure levels according to Guidelines for the Management of Hypertension set by the Japanese Society of Hypertension (JSH 2009) [16]. The exclusion criteria were as follows: secondary hypertension, DM, serum creatinine levels ≥ 2.0 mg/dL, symptomatic heart failure, acute cardiovascular diseases 3 months prior to the examination, history of gastrointestinal surgery, and systemic diseases, such as hepatic disease, collagen disease, and malignancy. None of the subjects changed their medications or daily dietary habits during the examination period. Subjects received full verbal and written explanations of the nature and purpose of this study and gave their written informed consent. The study was approved by the Ethical Committee of Juntendo University. This study has been registered in the UMIN Clinical Trials Registry System as the trial ID UMIN R000006809 and the abbreviated trial name as AGENT.

### Study design

**Figure 1:**
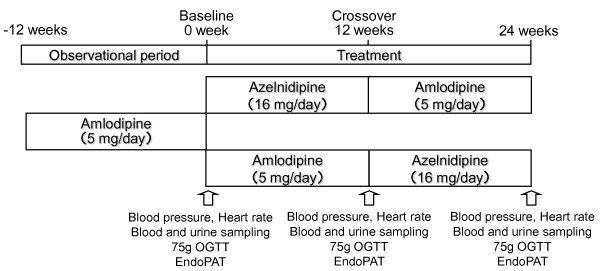
**Study design of this study**. OGTT; oral glucose tolerance test.

This study was a prospective randomized crossover design (Figure 1). Randomization was undertaken by a nontreating physician using a table of random numbers to match the two groups for age, sex, and glycated hemoglobin (HbA1c) levels. After randomization, azelnidipine 16 mg once daily or amlodipine 5 mg once daily was administered in a crossover manner each for 12-week.

### Blood and Urine Sampling and Measurements

At the beginning of this study and the end of each treatment, a blood sample was taken from an antecubital vein after 12 h of fasting. Plasma levels of total cholesterol (TC), triglyceride (TG), high-density lipoprotein cholesterol (HDL-C), and high-sensitivity C-reactive protein (hs-CRP) were measured using standard methods. Low-density lipoprotein cholesterol (LDL-C) levels were calculated using Friedewald's formula. The value for HbA1 c (%) was estimated as National Glycohemoglobin Standardization Program equivalent value (%) calculated by the formula: HbA1 c (%) = HbA1 c [Japan Diabetic Society (JDS)] (%) + 0.4%, considering the relational expression of HbA1 c (JDS) (%) as measured by the previous Japanese standard substance and measurement methods [17]. Plasma levels of IL-6 were measured by chemiluminescent enzyme immunoassay methods. The standard 75 g OGTT was performed in a fasting state. Blood samples for the measurement of glucose and insulin were drawn just before glucose administration, as well as 30, 60 and 120 min later, as previously described [3]. The morning urine sample was collected for urinalysis at the beginning and the end of each therapy period. Urinary albumin and L-FABP were measured by an immunoturbidimetric method and a specific enzyme-linked immunosorbent assay, respectively [10]. Both urinary values were expressed as ratios to the urinary creatinine concentration.

### Flow Cytometric Enumeration of Hematopoietic Progenitor Cells (HPCs)

Circulating number of HPCs were quantified with ProCount Progenitor Cell Enumeration Kit (BD Biosciences, San Jose, CA, USA) according to manufacturer's instructions using a FACS Canto II Flow Cytometer (BD, Franklin Lakes, NJ, USA) [18]. This assay has an inter-assay coefficient of variation of 7.0%.

### Measurement of Reactive Hyperemia by Peripheral Arterial Tonometry

Endothelial function as determined by reactive hyperemia in peripheral arteries was measured using fingertip pulse amplitude tonometry employing an EndoPAT 2000 device (Itamar Medical Inc., Caesarea, Israel), as previously described [19]. Briefly, finger probes were placed on the middle finger of each subject's hand. After a 5 min baseline measurement, the blood pressure cuff on the test arm was inflated to 60 mmHg above the baseline systolic blood pressure (SBP) or ≥ 200 mmHg for 5 min. After 5 min, the cuff was deflated and peripheral arterial tonometry (PAT) was recorded for further 6 min. The ratio of the PAT signal after cuff release compared with baseline value was automatically calculated through a computer algorithm normalizing for the baseline signal and indexed to the other arm.

### Statistical Analysis

Results are represented in the mean values ± SD. Statistical analyses were performed using SPSS software (Version 17.0, SPSS Japan Inc., Tokyo, Japan). We used two-way ANOVA to detect any significant differences, which were later evaluated by post hoc analysis (i.e., Tukey's test). Because this is a crossover study, two independent variables between azelnidipine and amlodipine administration were needed for analyses. *P *< 0.05 was considered to be significant.

## Results

### Patient characteristics

One patient was newly diagnosed as having DM after the 75 g OGTT. Therefore, analysis was performed on 17 subjects. Table 1 shows baseline patient characteristics. Of the 17 subjects, 11 were men (mean age 56 ± 13 years). Mean levels of HbA1 c were relatively low in the study subjects [5.5 ± 0.4% (JDS 5.1 ± 0.3%)].

**Table 1:** Baseline characteristics of patients

**Male/female**	11/6
**Age, years**	56 ± 13
**Body mass index, kg/m^2^**	24.4 ± 4.0
**Impaired glucose tolerance (%)**	7 (41)
**Dyslipidemia (%)**	8 (47)
**Current smoker (%)**	3 (18)
**History of CHD (%)**	1 (6)
**Hemoglobin A1c, % (JDS)**	5.5 ± 0.4 (5.1 ± 0.3)
**Fasting blood sugar, mg/dL**	96 ± 8
**Fasting IRI, μIU/mL**	6.9 ± 4.2
**eGFR, ml/min/1.73 m^2^**	83.2 ± 18.3
**Medications**	
**ACE-inhibitor (%)**	1 (6)
**ARB (%)**	4 (24)
**Beta-blocker (%)**	1 (6)
**Diuretics (%)**	0 (0)
**Statin (%)**	2 (12)

Data are mean ± SD or numbers. CHD; coronary heart disease, JDS; Japan Diabetes Society, IRI; immunoreactive insulin, eGFR: estimated glomerular filtration rate; ACE; angiotensin converting enzyme, ARB; angiotensin-receptor blockers.

### Blood pressure and heart rate

Table 2 shows the results of blood pressure and heart rate at baseline and after administration of the two medications. No significant differences were observed in SBP (120.2 ± 11.7 vs. 121.3 ± 8.6 mmHg, *P *= 0.492) and diastolic blood pressure (DPB) (66.8 ± 7.2 vs. 70.1 ± 8.4 mmHg, *P *= 0.145) after administration of azelnidipine and amlodipine, respectively. The heart rate after azelnidipine administration was significantly lower than that after amlodipine administration (60.5 ± 6.6 vs. 65.1 ± 7.6/min, *P *= 0.003).

**Table 2:** Comparison of blood pressure and heart rate between treatment with azelnidipine and amlodipine

	Baseline	Azelnidipine N = 17	Amlodipine N = 17	P **
**SBP (mmHg)**	119.9 ± 11.5	120.2 ± 11.7	121.3 ± 8.6	0.492
**DBP (mmHg)**	71.6 ± 10.6	66.8 ± 7.2	70.1 ± 8.4	0.145
**Heart rate**	64.9 ± 5.9	60.5 ± 6.6*	65.1 ± 7.6	0.003

Data are mean ± SD. SBP; systolic blood pressure, DPB; diastolic blood pressure. * *P *< 0.005 vs. baseline. ** azelnidipine vs. amlodipine.

### 75 g OGTT

Table 3 shows the levels of blood glucose and immunoreactive insulin (IRI) after the 75 g OGTT at baseline and after administration of the two medications. 120-min glucose levels after azelnidipine administration were significantly lower than those after amlodipine administration (130 ± 36 vs. 149 ± 30 mg/dL, *P *= 0.039). The levels of 120-min IRI after azelnidipine administration were significantly lower than those after amlodipine administration (47.7 ± 36.4 vs. 57.2 ± 37.9 μIU/mL, *P *= 0.026).

**Table 3:** Comparison of 75 g oral glucose tolerance test between treatment with azelnidipine and amlodipine

	Baseline	Azelnidipine N = 17	Amlodipine N = 17	P **
**Glucose 0, mg/dL**	96 ± 8	94 ± 7	94 ± 8	0.826
**Glucose 30, mg/dL**	174 ± 28	162 ± 34	172 ± 25	0.160
**Glucose 60, mg/dL**	172 ± 37	181 ± 48	171 ± 41	0.221
**Glucose 120, mg/dL**	137 ± 30	130 ± 36	149 ± 30	0.039
**IRI 0, μIU/mL**	6.9 ± 4.2	7.0 ± 3.0	7.0 ± 3.5	0.957
**IRI 30, μIU/mL**	39.7 ± 16.9	39.8 ± 21.1	42.4 ± 20.8	0.660
**IRI 60, μIU/mL**	55.9 ± 29.8	56.1 ± 26.4	51.8 ± 27.3	0.465
**IRI 120, μIU/mL**	44.0 ± 26.8	47.7 ± 36.4	57.2 ± 37.9	0.026

Data are mean ± SD. IRI; immunoreactive insulin. ** azelnidipine vs. amlodipine.

### Laboratory data and PAT ratio

Table 4 shows the results of blood laboratory data and PAT ratio at baseline and after administration of the two medications. Changes of TC, LDL-C, HDL-C, and HbA1 c showed no significant differences between each drug administration group. After azelnidipine administration, hs-CRP levels tended to be lower (0.36 ± 0.23 vs. 0.74 ± 0.76 mg/L, *P *= 0.067) and IL-6 levels were significantly lower (1.44 ± 0.73 vs. 1.81 ± 0.78 pg/L, *P *= 0.035) than those after amlodipine administration. Circulating numbers of HPC after azelnidipine administration were significantly higher than those after the amlodipine administration (3.56 ± 1.59 vs. 2.65 ± 1.17/μL, *P *= 0.016). No significant differences were observed in estimated glomerular filtration rate, L-FABP levels, or urinary albumin levels after the administration of azelnidipine or amlodipine. No significant differences in the PAT ratio were observed after administration of each of the drug. There were no abnormalities in blood chemistry or other clinical parameters during this study period. In addition, no adverse events were observed.

**Table 4:** Comparison of laboratory data and PAT ratio between treatment with azelnidipine and amlodipine

	Baseline	Azelnidipine N = 17	Amlodipine N = 17	P **
**TC, mg/dL**	191 ± 17	194 ± 22	191 ± 23	0.617
**LDL-C**, **mg/L**	109 ± 19	111 ± 24	110 ± 19	0.730
**HDL-C, mg/dL**	57 ± 17	58 ± 18	57 ± 17	0.437
**Triglyceride, mg/dL**	104 ± 60	110 ± 57	114 ± 65	0.750
**HbA1c, %**	5.1 ± 0.3	5.1 ± 0.2	5.1 ± 0.3	0.999
**hs-CRP, mg/L**	1.14 ± 2.66	0.36 ± 0.23	0.74 ± 0.76	0.067
**Interleukin-6, pg/mL**	1.31 ± 0.61	1.44 ± 0.73	1.81 ± 0.77	0.035
**eGFR, ml/min/1.73 m^2^**	83.2 ± 18.3	82.3 ± 16.1	79.4 ± 15.8	0.188
**L-FABP/U-Cr, ng/g Cr**	29.4 ± 40.0	13.9 ± 16.2	17.9 ± 16.9	0.187
**Micro-alb/U-Cr, mg/g Cr**	23.6 ± 47.4	26.6 ± 59.9	38.0 ± 87.4	0.120
**HPC,/μL**	2.79 ± 1.67	3.56 ± 1.59*	2.65 ± 1.17	0.016
**PAT ratio**	1.93 ± 0.34	2.05 ± 0.39	2.11 ± 0.47	0.587

Data are mean ± SD. TC; total cholesterol, LDL-C; low-density lipoprotein cholesterol, HDL-C; high-density lipoprotein cholesterol, Hb; hemoglobin, hs-CRP; high sensitivity C-reactive protein, BNP; brain natriuretic peptide, eGFR; estimated glomerular filtration ratio, GFR(ml/min/1.73 m^2^) L-FABP, L-type fatty acid binding protein, alb; albuminuria, U-Cre; urine creatinine, HPC; hematopoietic progenitor cell, PAT: peripheral arterial tonometry. * *P *< 0.05 vs. baseline. ** azelnidipine vs. amlodipine.

## Discussion

The adverse impact of new-onset DM in treated patients with essential hypertension is well established [6]. It is well known that renin-angiotensin system-related agents such as ACE inhibitors and ARBs have potential for preventing new-onset of DM [7,20]. However, the effect of CCBs on glucose tolerance and insulin sensitivity has not been clearly elucidated, particularly in the clinical setting. The present study demonstrated that azelnidipine administration rather than amlodipine administration significantly ameliorated glucose intolerance and the inflammatory state in non-diabetic patients with essential hypertension. In addition, the number of circulating HPCs was significantly higher after azelnidipine administration than those after amlodipine administration. This study is, to the best our knowledge, a first report that demonstrates the beneficial effects of azelnidipine on glucose tolerance and insulin sensitivity in non-diabetic patients with essential hypertension.

The reason why azelnidipine ameliorated glucose tolerance and insulin response in non-diabetic patients with essential hypertension should be discussed. Recent studies have clearly demonstrated that inflammation and oxidative stress play an important role in the pathogenesis of hypertension and/or DM as well as atherosclerosis [21-24]. Activated proinflammatory cytokines and increased oxidative stress elicit damage to various organs. Indeed, increased concentrations of proinflammatory cytokines induce a shift toward impaired glucose tolerance [23,25]. In the present study, circulating levels of IL-6 and hs-CRP were lower after azelnidipine administration than those after amlodipine administration. A previous study also demonstrated that azelnidipine significantly decreased plasma levels of monocyte chemoattractant protein-1, IL-6, hsCRP, TNF-α, 8-epi-prostaglandin F_2α_, and 8-hydroxydeoxyguanosine in patients with diabetic nephropathy [22]. Azelnidipine has been shown to prevent TNF-induced activation of endothelial cells and IL-8 expression via its antioxidative properties [12,26]. A recent study showed that azelnidipine even at a non-hypotensive dose improved glucose tolerance and superoxide production in the skeletal muscle of diabetic mice [14]. Although direct evidence regarding oxidative stress is not available in the present study, the anti-inflammatory effect of azelnidipine may, at least in part, contribute to the improvement of glucose tolerance. However, in the subjects of upper median L-FABP/U-Cr values at baseline, the levels of L-FABP/U-Cr after azelnidipine administration were significantly lower than those after amlodipine administration (data not shown). A recent study reported no significant changes in fasting glucose levels after azelnidipine administration [13]. Indeed, fasting blood glucose and HbA1 c levels did not show significant changes even after azelnidipine administration in the present study. However, 120-min glucose and insulin levels after azelnidipine administration were significantly lower than those after amlodipine administration. To evaluate any small changes in glucose and insulin metabolism, 75 g OGTT must be performed. Another possibility is inhibition of the sympathetic nervous system by azelnidipine treatment. Increased heart rate is a sign of the increased sympathetic activity [27]. Increased heart rate is associated not only with multiple coronary risk factors, but also morbidity and mortality of cardiovascular diseases [27]. Indeed, enhanced sympathetic tone could cause insulin resistance by β-adrenergic stimulation [27]. It has been reported that dihydropiridine CCBs, even third generation CCB, such as amlodipine, increase plasma norepinephrine levels and the ambulatory heart rate [28]. However, azelnidipine has been reported to prevent an increase in heart rate by inhibition of the sympathetic nerve center, rostral ventrolateral medulla [29]. After azelnidipine administration, the heart rate was significantly reduced and was significantly lower than that after amlodipine administration in the present study. Therefore, the anti-sympathetic nervous system effect of azelnidipine may contribute to a favorable effect on glucose tolerance.

Several studies have reported decreased numbers of circulating progenitor cells such as endothelial progenitor cells (EPCs) and HPCs in patients with CAD as well as in patients at high-risk of cardiovascular diseases [21,30]. In the present study, the number of circulating HPCs after azelnidipine administration was significantly higher than after amlodipine administration. Most previous studies investigated the number of EPCs with respect to atherosclerotic disorders. However, a recent study reported that the numbers of HPCs, rather than EPCs, were associated with endothelial dysfunction as assessed by an intracoronary acetylcholine challenge test [21]. Unfortunately, no internationally standardized set of criteria for EPC definition have yet been established. On the contrary, circulating HPCs are defined as CD34^+ ^and CD45^dim ^cells by the International Society of Hematotherapy and Graft Engineering [31]. Therefore, we measured the number of circulating HPCs using a standardized assay kit. A comparative study that measures the number of circulating EPCs using a standardized definition must be investigated in the future.

Vascular endothelial dysfunction contributes to the initiation and progression of arteriosclerosis and serves as a strong predictor of cardiovascular events [32,33]. We previously reported that slight elevations of glucose and insulin levels after 75 g OGTT are associated with the severity of CAD even in patients with normal glucose tolerance [3]. Ceriello *et al.* recently demonstrated that oscillating glucose levels have more deleterious effects than constant high glucose on endothelial function in diabetic patients [34]. We also reported that postprandial hyperglycemia as well as hyperlipidemia induces endothelial dysfunction [35]. In addition, improvement of postprandial hyperglycemia by α-glucosidase inhibitors prevents endothelial dysfunction in diabetic patients with CAD [35]. In the present study, no significant differences in endothelial function as assessed by the PAT ratio were observed after administration of the two drugs. Although the number of circulating HPCs was significantly increased after azelnidipine treatment, much longer time might be needed to improve endothelial dysfunction in this study population. Another possibility is the timing of endothelial function assessment. The changes in endothelial function at fasting may be relatively small compared with the changes in postprandial values [35,36]. Several non-invasive methods have been developed to measure endothelial function and the PAT ratio is one of the useful devices to assess endothelial function [19]. Further studies are needed to elucidate the association between endothelial function and differences in CCBs in the next step.

There were several potential limitations in the present study. First, the sample sized was small. We conducted a randomized crossover study. Therefore, we believe that the differences between azelnidipine and amlodipine are reliable. Second, we assessed the effects of administration of each CCB for 12 weeks. Investigation of the impact of long-term effects, including postprandial parameters and clinical outcomes, is needed in the future. Third, as described above, the mechanisms by which azelnidipine ameliorated glucose tolerance after 75 g OGTT in non-diabetic patients with hypertension, has not been clearly elucidated. Fourth, we demonstrated the data of the PAT ratio in endothelial function analyse, but several parameters can be used to assess endothelial function. Fifth, the reproducibility of 75 g OGTT may require further examinations. However, all testing procedures were performed under the same conditions, including those of the start time, room temperature, and quite waiting place.

## Conclusions

These results suggest that azelnidipine treatment may have beneficial effects against glucose intolerance, insulin sensitivity, the inflammatory state, and circulating numbers of progenitor cells in non-diabetic patients with essential hypertension. Further prospective investigations in a large population are required to confirm these findings.

## Competing interests

The authors declare that they have no competing interests.

## Authors' contributions

KF participated in planning of the study, recruiting study subjects, and analysis. KS contributed at all stages drafted the manuscript. MH and HO participated in HPCs and EndoPAT. TakaK, KH, AK, RM, TakeK, and TM involved in recruiting study subjects and discussing of results. HD contributed in planning of the experiment and discussion of results as well as supervising the study. All authors read and approved the final manuscript.
